# Study on the Enhancement Effect of Synergy between Multi-Size Functionalized Boron Nitride and Graphene Oxide on the Thermal Properties of Phase Change Composites

**DOI:** 10.3390/molecules28093797

**Published:** 2023-04-28

**Authors:** Song Xin, Zhiwen Zhao, Shangxiao Liu, Jiedong Liu, Mengya Li

**Affiliations:** 1 College of Transportation, Shandong University of Science and Technology, Qingdao 266590, China; 2 College of Safety and Environmental Engineering, Shandong University of Science and Technology, Qingdao 266590, China; 3 State Key Laboratory of Mining Disaster Prevention and Control Co-Founded by Shandong Province and the Ministry of Science and Technology, Shandong University of Science and Technology, Qingdao 266590, China

**Keywords:** phase change materials, boron nitride nanosheets, functionalization, thermal conductivity

## Abstract

Boron nitride nanosheet (BNNS) and graphene oxide (GO) as a single filler can effectively improve the thermal conductivity of the composites, and the synergistic mechanism of BNNS and GO was investigated in this paper. In this study, BNNS was first surface-functionalized and the multi-sized (50 nm, 200 nm, 500 nm) modified BNNS (A-BN) were attached to GO through non-covalent bonding interactions to form a cross-linked structure. Then, A-BN and GO were used as thermal fillers and support material adsorption eutectic phase change materials (PCMs) to prepare composite phase change material (CPCM). Characterization results show that small-size A-BN was more likely to form dense thermal networks with good compatibility and interface connectivity between PCMs, A-BN, and GO, ensuring that PCMs can be stored in the network without leaking. When the size of the BNNS was greater than 200 nm, the advantage of thermal conductivity obtained by A-BN was no longer obvious, and the phase change behavior of CPCM was inhibited. In general, the prepared CPCM has the ideal thermal response and thermal stability, which is very suitable for energy storage and thermal management applications.

## 1. Introduction

In recent years, the energy field has continued to cope with energy consumption through innovative technologies. As a functional material, phase change materials (PCMS) have the advantages of high energy storage density, low cost, and no pollution, and can absorb, store, and release large amounts of thermal energy in a specific temperature range [1,2,3], and are widely used in construction [4], road construction [5], textiles [6], electronic components [7], and other industries.

With the continuous development of PCMS, organic PCMS such as paraffin, alkanes, and alcohols are widely used because of their high latent heat, good thermal stability, and low cost. Among them, eutectic phase change materials with the lowest melting point formed by mixing two or more organic phase change materials in a certain ratio not only have the above advantages but can also effectively solve the problem of temperature limitations existing in a single system [8,9,10,11,12]. However, low thermal conductivity, poor shape stability, and susceptibility to leakage limit the scope of application of conventional organic PCMs. Encapsulation of PCMs using carbon-based materials and metal-based materials is an ideal route [13,14]. Zhang et al. [15] prepared n-octadecane/polystyrene/expanded graphite (EG) composites with a thermal conductivity of 1.01 W/(m∙K), seven times higher compared to PCMs. However, although EG possesses a strong adsorption capacity, its low thermal conductivity affects the phase change process of PCMs. Hexagonal boron nitride (h-BN) is a white compound with hexagonal layering similar to graphite, with better corrosion resistance, oxidation resistance, high thermal conductivity, insulation, and wave transport [16,17,18]. The stripped boron nitride nanoscale (BNNS), with less phonon scattering and a higher aspect ratio, is an ideal thermal filler [19,20,21]. For example, Qian et al. [12] prepared PCMs by infiltrating paraffin into h-BN porous scaffolds with continuous thermal conductivity paths, and when the h-BN content was 18 wt%, the latent heat of melting of the composites was 165.4 ± 1.7 J/g and the thermal conductivity reached 0.85 W/(m∙K), which was a six-fold increase in thermal conductivity compared to pure paraffin; Dimberu et al. [22] prepared paraffin/metal-organic gel/boron nitride composites by impregnation with a thermal conductivity of 232.1% of pure paraffin wax and a phase change latent heat of 163.1 J/g. Lei et al. [23] prepared PEG/cellulose/BNNS composites by vacuum impregnation and cold compression using the self-assembled properties of concentrated cellulose/BNNS aqueous blends, taking full advantage of the efficient orientation and high in-plane thermal conductivity of BNNS during cold compression, and obtained composites with a thermal conductivity of 4.764 W/(m∙K), which was about 14 times higher, and with good shape stability, maintaining their original shape at 101 °C.

Some scholars have pointed out that the thermal resistance between materials is mainly caused by inherent phonon spectral mismatch. Only the filler surface is functionalized, and the energy of lattice vibration is still reduced by differences in the acoustic properties and dispersed at the interfaces between materials [24,25,26,27,28,29]. Therefore, the degree of matching between fillers should be fully considered. The addition of carbon-structured substances highly conjugated with high thermal conductivity fillers can effectively solve the above problems. Graphene oxide (GO) has a high thermal conductivity, high specific surface area, and low density, and has a large number of oxygen-containing functional groups on its surface, which can play a synergistic role with h-BN by self-assembly to improve the thermal conductivity of the material, and its good dispersion and high surface area can encapsulate PCMs and prevent leakage of the material [30,31,32,33,34].

Through the understanding of the previous related research, lauric acid and n-octadecane eutectic not only have a high latent heat of phase change, but also after calculation and analysis, the eutectic temperature of lauric acid-n-octadecane eutectic is very suitable for the application of temperature control wall, cooling suit, and other fields. In this paper, an eutectic mixture of lauric acid (LA) and n-octadecane (ODE) was used as the PCM, GO was used as heat filler, BNNS was used as the heat conduction enhancement medium, and the surface functionalization of BNNS with different sizes was performed to improve the compatibility between BNNS and GO. The effect of a synergistic effect between GO and BNNS of different sizes on the phase change behavior and thermal conductivity of CPCM was investigated by using self-assembly to connect BNNS with GO, which provides an idea of how to improve the thermal storage capacity and thermal conductivity of CPCM.

## 2. Results and Discussion

### 2.1. Thermal Storage Properties of CPCM

**Figure 1:**
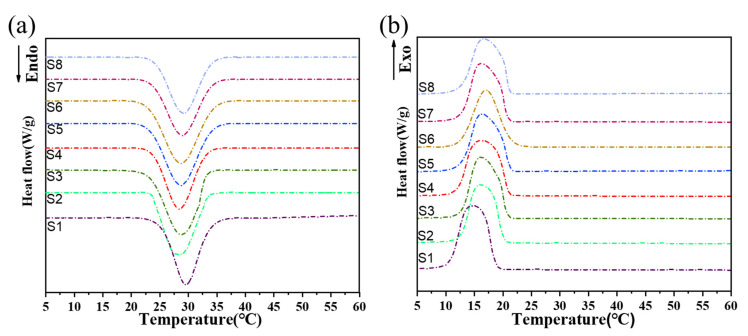
The DSC test curves of the LA-ODE and LA-ODE/A-BN/GO CPCMs: (**a**) melting process and (**b**) freezing process.

The thermal properties of the LA-ODE and LA-ODE/A-BN/GO CPCMs were tested using DSC, as shown in Figure 1, and the thermal performance data are recorded in Table 1. The melt and solidification temperatures of LA-ODE were 24.05 °C and 18.27 °C, respectively, which were 0.9 °C different from the previously calculated melt temperature (23.15 °C), which was very close to the theoretical prediction using Schroeder’s formula, and the error of the melt temperature was 3.8%, which was within the acceptable range. Meanwhile, the melting temperature of the CPCM reached the desired temperature range after adding different sizes of A-BN and different contents of GO. In the range of 10~45 °C, LA-ODE and the CPCM showed strong and sharp heat absorption and exothermic peaks during melting and solidification, which proved that the LA-ODE binary eutectic mixture had a high heat storage capacity and could still maintain good phase change properties even when adsorbed by GO. In addition, the thermal responsiveness of the material was improved after the addition of A-BN and GO, which was seen by a slight shift in the thermal property curve of the CPCM, and the melting temperature of the CPCM showed a decreasing trend with the increasing content of A-BN and GO. The latent heat of the phase change of the CPCM decreased after the addition of A-BN and GO. On one hand, the incorporation of A-BN and GO will occupy the storage space of PCMs and does not contribute to the latent heat of phase change of the composites. On the other hand, the large specific surface area of A-BN and GO enhanced the interaction with PCMs and overcame the van der Waals forces between the thermally conductive material and PCMs during the melting process, which led to a change in the molecular arrangement of PCMs and affected the free energy state and latent heat of the system [35]. Overall, the incorporation of A-BN and GO can improve the stability of CPCM, but the determination of its optimal incorporation amount and the synergistic effect between A-BN and GO of different sizes deserves further analysis.

### 2.2. Micromorphological Analysis of CPCM

**Figure 2:**
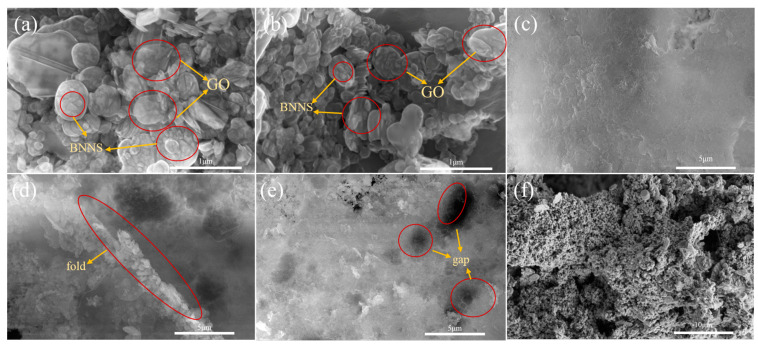
SEM images of (**a**–**c**,**f**) S3; (**d**) S7, and (**e**) S8.

To analyze the microstructure, filler dispersion, and network connections between fillers of the composites, the surface and spatial structure of the composites were observed by field emission scanning electron microscopy, and the resulting micrographs are shown in Figure 2. The results showed that A-BN was attached to the GO surface, and there were no obvious voids and agglomerations, indicating that the silane molecules of A-BN promoted the dispersion of A-BN. The good compatibility and interfacial connectivity between PCM, A-BN, and GO ensure that the PCMs can be stored in the network without leakage. Figure 2c shows that the addition of A-BN can make the composite surface uniformly smooth and dense, and the thermal conductivity network denser and more abundant. However, after the addition of a larger size of A-BN, an agglomeration phenomenon occurred and the edge of the thermally conductive filler was wrinkled, which to a certain extent will hinder the thermal expansion of the PCMs, as shown in Figure 2d,e, and voids appear, resulting in poor interfacial compatibility of the CPCM and higher thermal resistance, which inhibits the material’s heat transfer and affects the composite’s thermal conductivity.

### 2.3. Crystal Structure of CPCM

**Figure 3:**
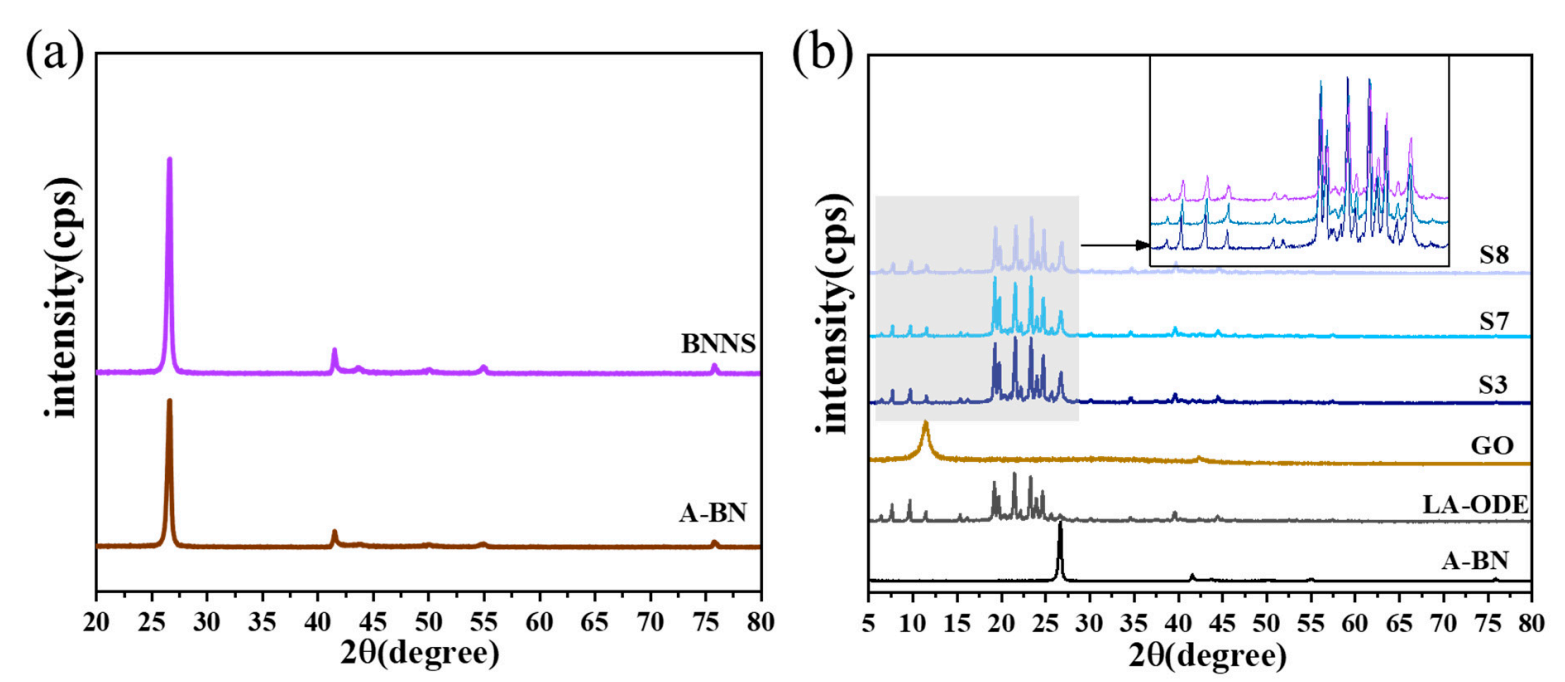
XRD patterns of (**a**) BNNS and A-BN; (**b**) XRD patterns of each component and CPCM.

XRD patterns were used to characterize the crystal structure of the composites, as shown in Figure 3. BNNS and A-BN showed six major characteristic diffraction peaks of different intensities at 2θ of 26.7°, 41.6°, 43.8°, 50.1°, 55.1°, and 75.9°, corresponding to the (002), (100), (101), (102), (004), and (100) crystal plane reflections, respectively, demonstrating that both BNNS and A-BN have a typical hexagonal crystal structure. Compared with LA-ODE, the positions of the characteristic diffraction peaks in the CPCM pattern were almost unchanged, indicating that both have similar crystal structures as well as cell types. The intensity of the characteristic diffraction peaks of the CPCM at 2θ for 26.7° and 11.5° was significantly reduced, indicating that the lamellar structure of A-BN and GO had an effect on the crystalline reflection of LA-ODE, which also indicates, to a certain extent, that A-BN and GO can play a good role in enhancing the adsorption effect ability. With an increase in the size of A-BN, the half-height width of the diffraction peak also widened, which indicates that the crystallinity of the CPCM was somewhat affected [36,37]. The above analysis shows that the addition of A-BN and GO did not affect the crystal structure of the CPCM, and the PCMs can still maintain good phase changeability.

### 2.4. Chemical Structure of CPCM

**Figure 4:**
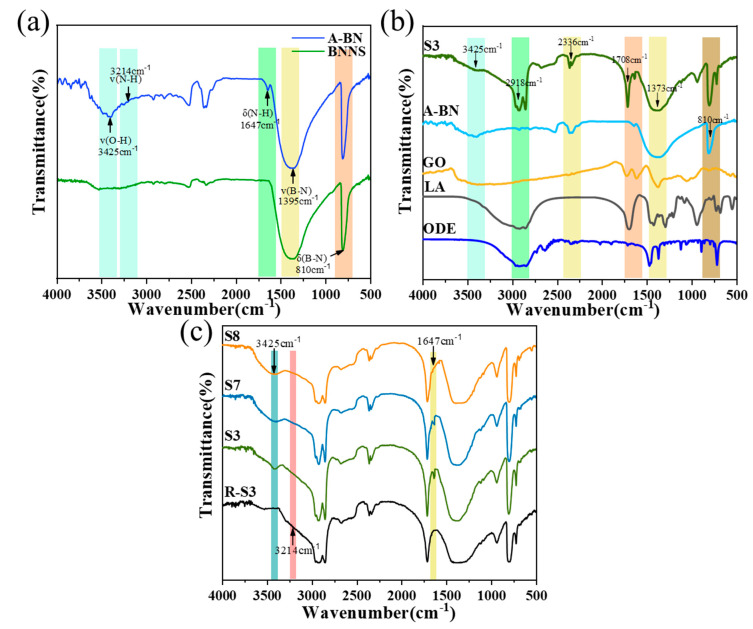
FTIR transmittance spectra of (**a**) BNNS and A-BN; (**b**) individual components and CPCM; (**c**) CPCM with different sizes of A-BN added.

To demonstrate the successful access of A-BN to the hydroxyl and amino groups, FTIR spectrograms of BNNS, A-BN, and the composites were studied. As shown in Figure 4a, the stronger characteristic peaks of BNNS and A-BN near 810 cm^−1^ and 1395 cm^−1^ corresponded to the in-plane stretching vibration mode with the B–N bond, and the out-of-plane bending vibration mode with the B–N–B bond, respectively. A-BN showed new characteristic peaks near 1647 cm^−1^, 3214 cm^−1^, and 3425 cm^−1^, corresponding to the stretching vibration mode and bending vibration mode of the N–H bond and the stretching vibration mode of the O–H bond [38,39], indicating the successful grafting of the hydroxyl group as well as the amino group of APTES on the surface of A-BN. As shown in Figure 4b, the two stronger characteristic peaks of GO appeared at 1645 cm^−1^ and 3396 cm^−1^, corresponding to the C–O stretching vibration and the O–H stretching vibration, respectively, which indicate the presence of a series of oxygen-containing functional groups such as the hydroxyl, carboxyl, and epoxy groups on the GO surface. Interestingly, the LA in the composite was significantly weakened at the C–O stretching vibration (939 cm^−1^), C=O stretching vibration (1704 cm^−1^), and methyl bending vibration of ODE (718 cm^−1^), indicating that LA-ODE is effectively encapsulated by the cross-linked structure generated by the support material through synergistic interaction, which can effectively prevent its leakage. Figure 4c exhibits the IR spectra of the composites composed of different sizes of A-BN, and it was found that S3, S7, and S8 showed new characteristic peaks near 1647 cm^−1^, 3214 cm^−1^, and 3425 cm^−1^, proving the successful surface functionalization of BNNS contained in the three composites.

**Figure 5:**
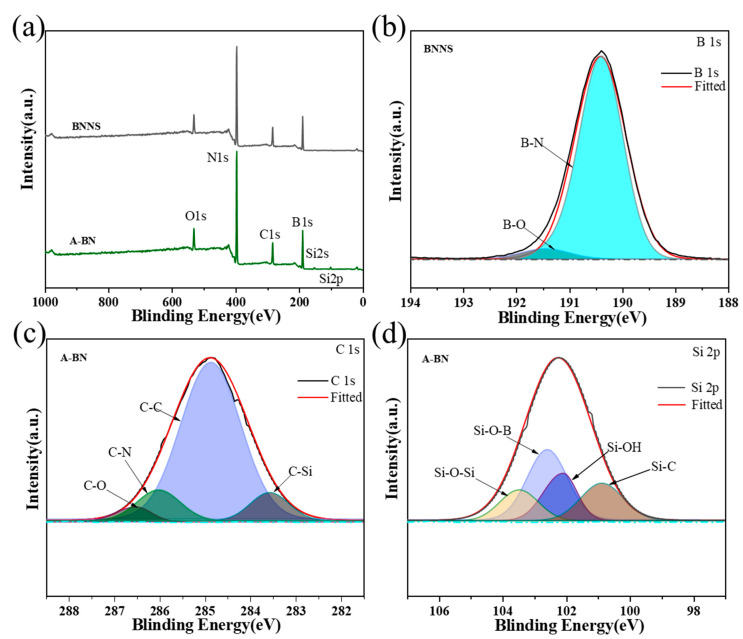
(**a**) XPS of the survey scan; (**b**) XPS B 1s spectra of BNNS; (**c**) XPS C 1s spectra of A-BN; (**d**) XPS Si 2p spectra of A-BN.

To further observe the modification of BNNS, the chemical structures of BNNS and A-BN were analyzed by X-ray photoelectron spectroscopy. Figure 5a,b shows that BNNS was mainly composed of B and N elements, the O element atoms and the weak B–O peak at 191.4 eV originated from the trace hydroxyl group carried by BNNS itself, and the C element originated from the particle-loaded carbon band in the test. Figure 5a,c,d shows that the content of the O element increased after BNNS was functionalized, which was caused by the generation of more hydroxyl groups on the A-BN surface. BNNS showed an increase in the C element and two additional peaks of the Si element after grafting with APTES. The C 1s spectrum of A-BN showed four fitted peaks of C–C, C–N, C–Si, and C–O at 248.8 eV, 286.1 eV, 286.6 eV, and 283.6 eV, respectively, and the Si 2p spectrum showed four fitted peaks of Si–O–B, Si–O–Si, Si–C, and Si–OH at 102.5 eV, 103.5 eV, 100.8eV, and 102.1eV, respectively. In combination with the IR pattern, the appearance of the fitted peaks and the Si elemental peaks proved that the hydroxylation of BNNS and the grafting with APTES were successful.

### 2.5. Thermal Stability of CPCM

**Figure 6:**
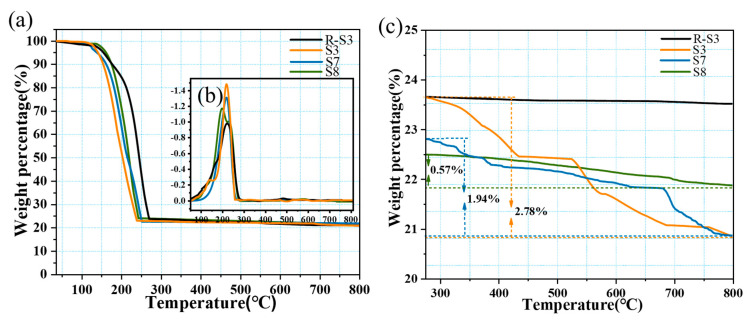
Thermal stability curves of the composites: (**a**) TG curves, (**b**) DTG curves, and (**c**) TG curves for R-S3, S3, S7, and S8 at 300−800 °C.

The thermal stability of CPCM occupies a very important position in practical applications. Figure 6 shows the TG curve and the DTG curve of the CPCM. The TG curves and DTG curves of the four samples showed the same trend, indicating that the behavior of functionalization of the BNNS surface does not affect the thermal performance of PCMs with good thermal stability, expanding the range of practical application scenarios. It was further observed that the thermal degradation rates of both S3 and S7 were greater than those of R-S3 and S8. S3 started thermal degradation when the temperature reached nearly 121.2 °C, and the heat loss of S3 was 50% when the temperature reached nearly 200.4 °C, which was caused by the lower heat transfer efficiency due to the lack of a good thermal conductivity network inside R-S3 and S8. The mass loss shown in Figure 6c corresponds to the mass loss of hydroxyl oxidation and silane molecules, and almost no mass loss occurred for R-S3, proving the good thermal stability of BNNS. The functionalized S3, S7, and S8 mass losses were 2.78%, 1.94%, and 0.57%, respectively, which also proved that the modification behavior was effective.

### 2.6. Thermal Conductivity of CPCM

**Figure 7:**
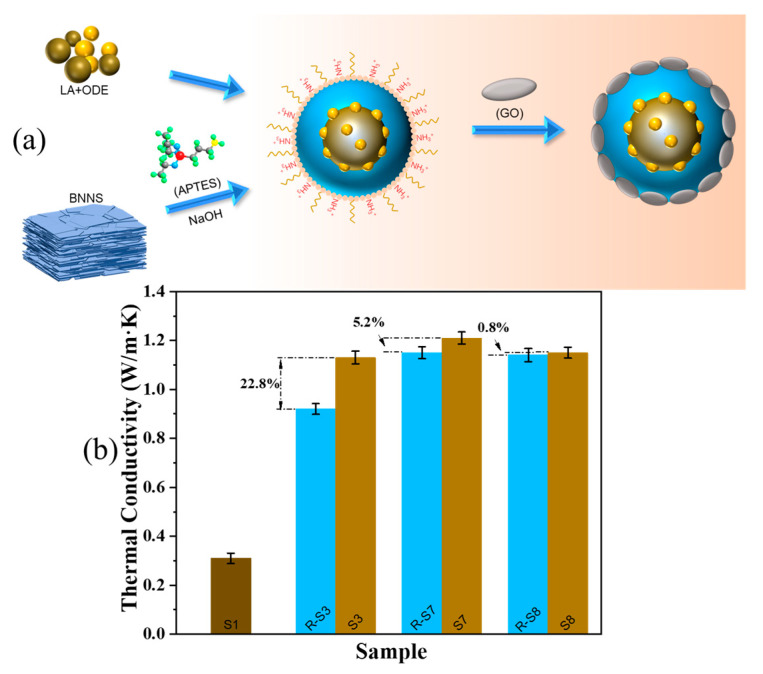
(**a**) Schematic diagram of the thermal conductivity enhancement mechanism of CPCM; (**b**) thermal conductivity of PCMs and different CPCM samples.

As shown in Figure 7a of the schematic diagram of the CPCM thermal conductivity enhancement mechanism, the synergistic effect of A-BN and GO can effectively wrap S1 and make up for its poor thermal conductivity. From Figure 7b, it can be derived that the thermal conductivity of S1 was low at 0.31 W/(m∙K), and after adding 12% BNNS and 2% GO, the thermal conductivity of sample R-S3 reached 0.92 W/(m∙K), which was 197% higher than S1. The thermal conductivity of S3 reached 1.13 W/(m∙K), which was 265% higher than that of S1, indicating that a good continuous dual matrix thermal conductivity network can be formed between A-BN and GO to improve the thermal response rate of the PCMs. The thermal conductivity of S8 was 1.15 W/(m∙K), which was 4.9% lower than that of S7. This is because when the size of A-BN is too large, it is easier to form a thermal conductivity network, but BNNS will create a certain gap when forming a thermal conductivity path, which will hinder the phonon transfer rate and increase phonon scattering [40,41,42,43].

### 2.7. Cyclic Stability of CPCM

**Figure 8:**
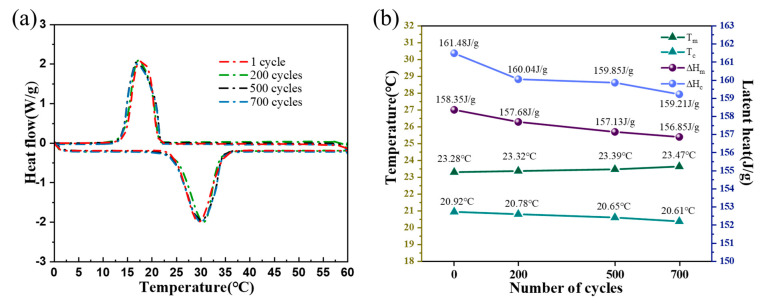
(**a**) Thermal cycling test data of S3; (**b**) latent heat, melting, and freezing points of S3 after thermal cycling.

To understand the material’s thermal stability, S3 was subjected to 200, 500, and 700 thermal cycles by differential scanning calorimetry, and the thermal performance data after the cycles were recorded in Table 2. The melting temperatures of S3 increased by 0.17%, 0.47%, and 0.81%, and the latent heat of melting decreased by 0.42%, 0.77%, and 0.94% after 200, 500, and 700 cycles of experiments, respectively. As shown in Figure 8, the melting and solidification temperatures kept fluctuating within a certain range during the cycling process, the area of the phase change peak was stable, and only one exothermic peak and one absorption peak appeared, indicating that the parameters of S3 can remain stable during the cycling process. Therefore, with the support of A-BN and GO, the PCMs have no leakage and still maintain good heat storage and release capacity, and have a long service life in practical applications.

## 3. Materials and Methods

### 3.1. Materials

Lauric acid (LA, C_11_H_23_COOH, AR, 99%) was purchased from Usolf Chemical Technology Co. Ltd. (Qingdao, China); 3-aminopropyltriethoxysilane (APTES), anhydrous ethanol, n-octadecane (ODE, C18H38, AR, 98%), graphene oxide (GO) were from Shanghai Maclean Biochemical Technology Co. Ltd.; boron nitride nanosheets (BNNSs) were purchased from Aladdin Chemical Co. Ltd. (Shanghai, China). All reagents were analytical and were not further purified.

### 3.2. Determination of the Ratio of the LA-ODE Binary Eutectic Mixture

The eutectic point temperature and optimal mixing ratio of the multivariate eutectic mixture PCMs were calculated by Schroeder’s Formula (1) [44].

**Figure 9:**
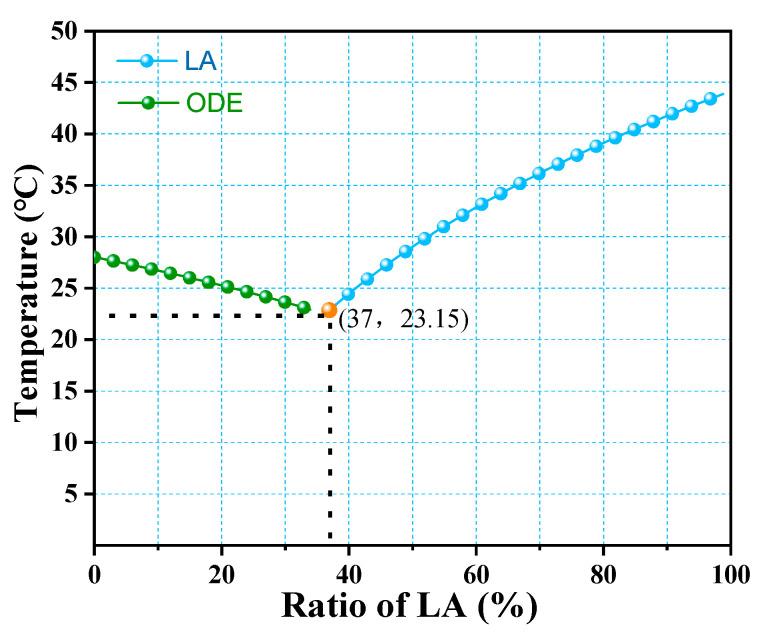
Theoretical phase diagram of the LA-ODE binary eutectic mixture.

Schroeder’s formula is as follows:

(1) $$T=\frac{H_{i}}{\frac{H_{i}}{T_{i}}-R\ln X_{i}},i=A,B$$

where T is the eutectic point temperature (K); $H_{i}$ is the molar heat of melting (J/mol) of component i at the eutectic point temperature; $T_{i}$ is the melting point (K) of component i. $X_{i}$ is the molar ratio of component i in the mixture and R is the gas constant (8.314 J/(mol·K)). The theoretical binary phase diagram of the predicted binary eutectic mixture is shown in Figure 9. The eutectic point is reached when the theoretical molar ratio of LA and ODE is LA:ODE = 37:63, and the eutectic point temperature is 296.15 K (23.15 °C).

### 3.3. BNNS Surface Functionalization

**Figure 10:**
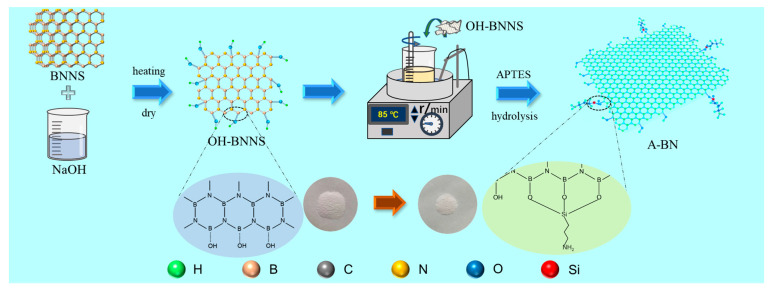
The A-BN preparation process.

**Figure 11:**
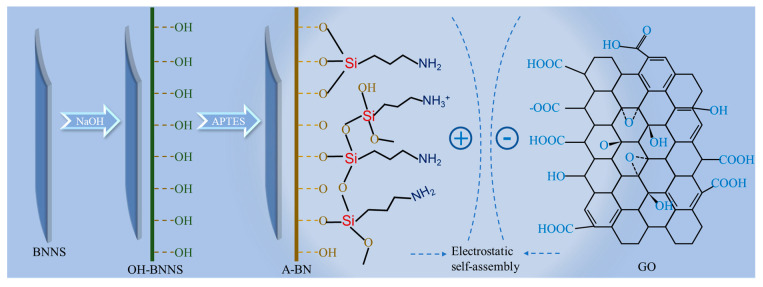
The surface functional group schematic diagram of A−BN.

As shown in Figure 10, BNNS was surface hydroxylated in a NaOH solution at a concentration of 5 mol/L, and then 2 mL of APTES and 1.2 g of BNNS were added to an aqueous ethanol solution (ethanol:water = 9:1) and stirred at 85 °C for 12 h to obtain APTES-BNNS (A-BN). The synergistic mechanism of A-BN and GO is shown in Figure 11.

### 3.4. Preparation of CPCM

**Figure 12:**
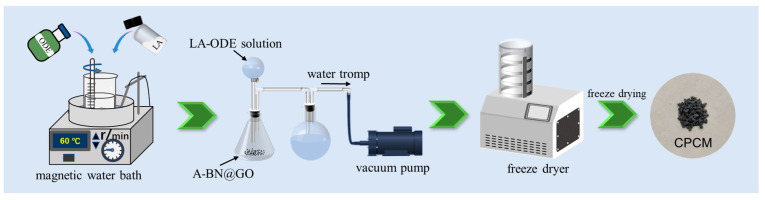
Preparation process of the CPCM.

The vacuum impregnation method was used to prepare the CPCM, as shown in Figure 12. First, LA (2.61 g), and ODE (6.09 g) were added to a constant temperature water bath heated pot, the temperature was set to 60 °C and stirred, and then mixed well and placed in a dispensing funnel. The A-BN@GO mixture was prepared by adding A-BN (1.2 g), and GO (0.1 g) using the same heating procedure, and then the A-BN@GO mixture was placed in a conical flask with the atmospheric pressure inside the flask pumped to 0.01 MPa. After the impregnation was completed, the vacuum pump was turned off to allow air to enter the flask again to ensure that the PCM was completely impregnated into the porous structure of A-BN@GO, and finally dried by a freeze-dryer. Sample S2 was obtained to determine the optimal ratio of GO in CPCM and to analyze the effect of multi-size surface functionalized BNNS on the thermal properties of the composites. According to this method, eight types of CPCM were prepared, named S1~S8, respectively, and the masses used for each component are shown in Table 3, and then tested by DSC. In addition, CPCM prepared using BNNS without modification treatment was used as a control group, named R-S3, and a total of four groups of materials, R-S3, S3, S7, and S8, were selected as the study subjects for other tests.

### 3.5. Characterization

The phase change temperature and phase change latent heat of CPCM were determined by a differential scanning calorimeter (DSC, Mettler DSC1, Mettler-toledo, Zurich, Switzerland) at a heating and cooling rate of 5 °C/min, the morphology of CPCM was characterized by scanning electron microscope (SEM, Apreo S Hivac, Thermo Fisher Scientific, Shanghai, China), and the surface functional groups of the observed CPCM were analyzed by Fourier infrared spectroscopy (FTIR, Nicolet IS50, Thermo Fisher Scientific, Shanghai, China) with a scan range of 500~4000 cm^−1^. Analysis of the crystal structure of CPCM was conducted by an X-ray diffractometer (XRD, Rigaku Utima, Rigaku corperationCity, Tokyo, Japan) with scanning diffraction angles of 5~80°; analysis of the element composition of A-BN was by an X-ray photoelectron spectrometer (XPS, ESCALAB250, Waltham, MA, USA); analysis of the thermal stability of CPCM at 30–800 °C was conducted by using a thermogravimetric analyzer (Mettler Toledo, Zurich, Switzerland) with a heating rate of 5 K/min; analysis of the thermal conductivity of CPCM was carried out by a heat disk thermal constant analyzer (TPS 2500, HotDisk, Gothenburg, Sweden). All measurements were performed in a nitrogen atmosphere.

## 4. Conclusions

In this study, BNNS was surface functionalized twice to enhance the interfacial compatibility and connectivity between BNNS and GO, and then the LA-ODE/A-BN/GO CPCM was prepared by the vacuum impregnation method. The structure, thermal storage properties, thermal conductivity, and thermal stability of CPCM were analyzed.

(1) The addition of A-BN and GO improved the thermal responsiveness of CPCM. With the addition of A-BN (50 nm) and GO at 12 wt% and 2 wt%, respectively, the latent heat of melting and the latent heat of solidification of CPCM could reach 158.35 J/g and 161.48 J/g, and the thermal conductivity reached 1.13 W/(m∙K), which was 265% higher than that of LA-ODE. CPCM had an ideal thermal stability, and the thermal properties of CPCM could still be maintained well after 700 thermal cycles.
(2) The microstructure shows that the small size of A-BN means that it is easier to form a dense thermal conductivity network, and the good compatibility and interfacial connectivity between PCMs, A-BN, and GO ensure that the PCMs can be stored in the network without leakage. The XPS, FTIR, and XRD characterization results showed that BNNS successfully grafted the hydroxyl group and APTES, and the addition of A-BN with GO did not affect the crystal structure of the PCMs.
(3) The thermal conductivity of CPCM with the addition of A-BN (50 nm) improved by 22.8% compared to R-S3, but the increase in thermal conductivity gradually decreased with the increase in size. The advantage of thermal conductivity gained by A-BN was no longer obvious when the size of A-BN was larger than 200 nm, indicating that the enhancement of thermal conductivity by the modification behavior is limited and there is a threshold value. This study can provide some help for the development of PCMs from other 2D nanosheet materials.

## Figures and Tables

**Table 1:** Thermal performance data of the PCMs and CPCMs in the heat absorption and exothermic processes.

Sample	Composite PCMs	Melting		Crystallization		$\Delta T\;({}^{\circ }C)$
		$T_{m}\;\left({}^{\circ }C\right)$	${\Delta H}_{m}\;\left(J/g\right)$	$T_{c}\;\left({}^{\circ }C\right)$	${\Delta H}_{c}\;\left(J/g\right)$	
S1	LO(LA-ODE)	24.05	186.45	18.27	181.62	5.78
S2	$\mathrm{LO}+{A\text{-}\mathrm{BN}}_{50\mathrm{nm}}+{\mathrm{GO}}_{1\%}$	23.64	166.27	20.45	163.24	3.19
S3	$\mathrm{LO}+{A\text{-}\mathrm{BN}}_{50\mathrm{nm}}+{\mathrm{GO}}_{2\%}$	23.33	158.35	20.84	161.48	2.49
S4	$\mathrm{LO}+{A\text{-}\mathrm{BN}}_{50\mathrm{nm}}+{\mathrm{GO}}_{3\%}$	23.28	151.53	20.92	153.63	2.36
S5	$\mathrm{LO}+{A\text{-}\mathrm{BN}}_{50\mathrm{nm}}+{\mathrm{GO}}_{4\%}$	23.14	148.42	21.11	143.58	2.03
S6	$\mathrm{LO}+{A\text{-}\mathrm{BN}}_{50\mathrm{nm}}+{\mathrm{GO}}_{5\%}$	23.10	142.34	21.21	146.21	1.89
S7	$\mathrm{LO}+{A\text{-}\mathrm{BN}}_{200\mathrm{nm}}+{\mathrm{GO}}_{2\%}$	23.37	160.27	20.75	159.24	2.62
S8	$\mathrm{LO}+{A\text{-}\mathrm{BN}}_{500\mathrm{nm}}+{\mathrm{GO}}_{2\%}$	23.45	163.24	20.52	160.15	2.93

**Table 2:** The rate of change of the latent heat of the melting and solidification of S3 after thermal cycling.

Number of Cycles	Melting				Crystallization			
	*T_m_* (°C)	Percentage Change (%)	Δ*H_m_* (J/g)	Percentage Change (%)	*T_c_* (°C)	Percentage Change (%)	Δ*H_c_* (J/g)	Percentage Change (%)
1	23.28	n.a	158.35	n.a	20.92	n.a	161.48	n.a
200	23.32	0.17	157.68	−0.42	20.78	−0.66	160.04	−0.89
500	23.39	0.47	157.13	−0.77	20.65	−1.29	159.85	−1.01
700	23.47	0.81	156.85	−0.94	20.61	−1.48	159.21	−1.41

n.a—no value.

**Table 3:** Mass of each component in the sample.

Sample	LA (g)	ODE (g)	A-BN (g)	GO (g)	Total (g)
S1	3.00	7.00	0	0	10
S2,	2.61	6.09	1.2	0.1	10
S3, S7, S8	2.58	6.02	1.2	0.2	10
S4	2.55	5.95	1.2	0.3	10
S5	2.52	5.88	1.2	0.4	10
S6	2.49	5.81	1.2	0.5	10

## Data Availability

Not applicable.
